# Gastrointestinal Infiltration in Influenza Virus Infection: Mechanisms and Clinical Insights

**DOI:** 10.3390/v17091187

**Published:** 2025-08-29

**Authors:** Aakriti Dua, Bhavna Trehan, Shymaa E. Bilasy, Catherine Yang, Ahmed ElShamy

**Affiliations:** 1 College of Medicine, California Northstate University, 9700 West Taron Drive, Elk Grove, CA 95757, USA; aakritidua05@gmail.com (A.D.); bhavna3641@gmail.com (B.T.); 2 College of Dental Medicine, California Northstate University, 2200 X Street, Sacramento, CA 95818, USA; shymaa.bilasy@cnsu.edu; 3 College of Graduate Studies, California Northstate University, 9700 West Taron Drive, Elk Grove, CA 95757, USA; catherine.yang@cnsu.edu

**Keywords:** influenza, lung–gut axis, gastrointestinal symptoms, immune dysregulation, inflammation, viral pathogenesis

## Abstract

**Background**: Influenza, a primarily respiratory illness, frequently manifests with gastrointestinal (GI) symptoms including nausea, vomiting, diarrhea, and abdominal pain. In this review, we analyze mechanisms describing GI infiltration and subsequent involvement of the GI system during influenza infection. Direct mechanisms involve the presence of viral particles in the GI tract and binding to sialic acid receptor, α2,3 and α2,6 linkages. The influenza virus may gain access to gut tissue via swallowing of respiratory secretions, hematogenous spread, or lymphocytic migration via the lung–gut axis. Indirect mechanisms involve immune system dysregulation via cytokine, interferon, and leukotriene flux, upregulation of consequential apoptotic pathways, or gut microbiome dysbiosis. Together, they promote secondary GI opportunistic infections. These findings improve our knowledge of GI infiltration during influenza infection which may aid in effective clinical diagnosis and treatment, ultimately improving patient outcomes.

## 1. Introduction

Influenza, a contagious viral illness caused by a negative-sense single-stranded RNA virus from the Orthomyxoviridae family, typically manifests with upper respiratory symptoms including cough, fever, myalgia, and congestion, with severe infections progressing to secondary pneumonia. The virus replicates in the epithelium of the upper and lower respiratory tracts, causing inflammation, erythema, and mucosal discharge [1]. In the US, seasonal flu incidence ranges from 5% to 20% [2]. The Centers of Disease Control and Prevention (CDC) estimates 40 million cases, 470,000 hospitalizations, and 28,000 deaths related to influenza viral infection in the 2023–2024 season [3]. Globally, the World Health Organization (WHO) estimates 290,000 to 650,000 annual deaths from influenza infection, without accounting for influenza-associated mortality from comorbid conditions like cardiovascular disease [4].

Influenza viruses affecting humans are classified into three main types: A, B, and C. Influenza A virus (IAV) and influenza B virus (IBV) are commonly associated with seasonal flu outbreaks, which are further characterized according to their hemagglutinin (HA) and neuraminidase (NA) surface glycoproteins [1]. Influenza C virus (ICV), a milder cause of lower respiratory infection in children less than 2 years of age, is characterized by hemagglutinin-esterase-fusion (HEF) protein instead of distinct HA and NA markers [1]. However, the global seropositivity of ICV antibodies in adults is more than 50%, indicating high rates of ICV infection [5]. Influenza D virus (IDV), an additional serotype identified in other mammals and swine, has not been recorded to infect humans [6,7].

Accumulating evidence indicates that influenza patients present with gastrointestinal (GI) symptoms, including nausea, vomiting, diarrhea, and abdominal pain. Nevertheless, the pathophysiological mechanism of GI involvement is poorly understood. In this review, we comprehensively searched PubMed, Google Scholar, and ScienceDirect to extract articles analyzing the viral impact on the GI tract. This study will provide a foundation for future investigations into the topic.

## 2. Discussion

### 2.1. Kinetics of Infection

The influenza virus gains access to the host cells in four distinct steps: (1) recognition of sialic acid (SA) receptor on the host cell by the viral HA receptor binding domain, (2) formation of an endosome encapsulating the viral particle, (3) translocation of the endosome to the nuclear membrane, and (4) fusion with the nuclear membrane via HA glycoprotein, causing the release of viral genome into the host nucleus for replication [8,9]. Inside the nucleus, viral replication is carried out by the viral RNA-dependent RNA polymerase. Viral replication is rapid; it takes 6 hours for IAV progeny to be released by the cell and 5 hours for viral proliferation, with each infected cell potentially infecting another 22 host cells [10].

### 2.2. Prevalence and Impact of GI Symptoms in Influenza Infection

GI symptoms, including diarrhea, vomiting, abdominal pain, and anorexia, often demarcate more severe manifestations of influenza infections, particularly those caused by IAV. These complications were more pronounced in children, elderly, and immunocompromised patients. IAV infection can be associated with more severe complications like appendicitis, abdominal pain, and hemorrhagic gastritis (Table 1) [11]. Influenza has also been implicated in mimicking acute abdomen, leading to a misdiagnosis with other GI, gynecological, or genitourinary etiologies [12,13].

Cases of influenza infection with associated GI symptoms have been previously reported in the literature. In a retrospective study performed on 627 patients with gastroenteritis, IAV RNA was detected in six hospitalized patients [14]. All six patients exhibited concurrent respiratory and GI symptoms, and subsequently, one patient died. In other patients, GI symptoms were present without concomitant respiratory symptoms. For instance, a patient presented to a Thailand hospital with isolated GI symptoms, including fever, diarrhea, nausea, and vomiting, and tested positive for influenza [15]. Another case involved a four-year-old boy with severe diarrhea who progressed to coma, encephalitis, and death without respiratory symptoms who had influenza A H5N1 virus isolated from his cerebrospinal fluid, serum, and throat and rectal swabs. Notably, his nine-year-old sister had died with similar symptoms two weeks earlier, although no diagnostic specimens were obtained and cause was not determined [16]. An immunocompromised four-year-old child diagnosed with influenza, presenting both respiratory and GI symptoms (non-bloody emesis and loose, watery diarrhea), had prolonged viral shedding in stool samples that persisted for >2 months from initial diagnosis [17]. Another two cases presented with abdominal symptoms such as pain and distention before the onset of respiratory influenza symptoms, and both GI and respiratory symptoms resolved with the use of Oseltamivir [12]. Owing to the severity of the GI symptoms, one of these two cases, a 66-year-old woman, underwent surgical exploration for a suspected colonic obstruction before H1N1 was diagnosed and Oseltamivir was initiated [12]. Another study that included 733 pediatric patients with diarrhea and concurrent influenza-like symptoms reported that 100 patients were positive for either IAV or IBV [18].

Influenza has been associated with hematemesis and hematochezia. During the 1988 H1N1 epidemic, seven children developed hematemesis with endoscopy revealing hemorrhagic gastritis [19]. A 21-year-old male with IAV-related hemorrhagic colitis presented with abdominal pain, diarrhea, and hematochezia with colonoscopy revealing inflammatory cell infiltration and ulcers in the descending and sigmoid colon [20]. Ischemic colitis, colonic apoptosis, and ileal ischemia following influenza were also reported, with some reporting severe complications such as ileal resection via laparotomy or fatal total organ failure [21,22,23]. A 42-year-old female, positive for IAV, initially presented with gastroenteritis but acutely progressed to ileal ischemia and organ failure due to fulminant hemophagocytic lymphohistiocytosis [22].

Appendicitis is a rare complication of influenza infection with increased incidence in children and young adolescents [11]. A 14-year-old female, positive for H1N1 virus, presented with abdominal pain, diarrhea, loss of appetite, fever and developed acute appendicitis, post-infectious encephalopathy, as well as duodenal perforation [24]. Similarly, case reports for a 9-year-old and a 15-year-old patient discussed the development of acute appendicitis concurrent with influenza A H1N1 infection, leading to emergent appendectomy [25,26]. These cases outline an interesting potential connection between H1N1 and appendicitis, though the mechanism remains unclear.

Taken together, several reports documented concurrent GI symptoms with influenza infections, which can range from mild diarrhea to life-threatening complications. Implementing comprehensive diagnostic procedures and using appropriate therapeutic measures may improve the patient’s outcome. IAV was the most frequently reported culprit for GI manifestations in reviewed studies. In contrast, information regarding the association of IBV and ICV with GI manifestation was limited, with only one case reported by Dilantika et. al. discussing coinfection with IAV and IBV. Additionally, most reported studies were observational case reports that concluded the correlation between GI manifestation and influenza infection. Therefore, future research should focus on the exact mechanism underlying GI symptomology in influenza infections.

**Table 1:** Case reports on major gastrointestinal symptoms associated with influenza.

Citation	DOI	Age/Gender	GI Complaint	Diagnostic Method	Influenza Type	Summary	Outcome
[12]	10.3396/ijic.v7i4.7855	66 y/o female, 65 y/o male	Severe abdominal pain, mimicking acute abdomen	H1N1 viral culture	A (H1N1)	2 cases initially presenting with acute abdominal pain; later confirmed H1N1	Both recovered with oseltamivir
[13]	10.1111/irv.12222	17 y/o female	Severe abdominal pain, vomiting	RT-PCR (nasopharyngeal)	A (H1N1)	Initially presented with worsening abdominal pain and nausea with increased pain in the right lower quadrant with rebound tenderness concerning for appendicitis; Abdominal CT, ultrasound, gynecologic, and genitourinary workup nondiagnostic. Influenza confirmed by PCR	Recovered with supportive care
[14]	10.1016/j .jcv.2009.06.011	Range: >1–97 y/o, all genders	Diarrhea, vomiting, gastroenteritis	RT-PCR of nasopharyngeal and fecal samples	A (H3N2)	Fecal detection of influenza A virus in 6/627 hospitalized patients with GI symptoms and confirmed respiratory flu	Recovered; virus detected in stool in subset
[15]	10.3201/eid1007.040415	39 y/o female	Vomiting, diarrhea, nausea	RT-PCR and viral culture	A (H5N1)	Mild case of avian influenza with GI symptoms	Recovered
[16]	10.1056/NEJMoa044307	4 y/o male	Watery diarrhea	RT-PCR and virus isolation	A (H5N1)	Fatal pediatric case with GI onset and encephalopathy.	Death
[17]	10.3201/eid1607.091248	4.5 y/o male	Prolonged viral shedding in stool	RT-PCR (stool)	A (H1N1)	Immunocompromised child with persistent influenza A RNA in stool for >1 month	Recovered; monitored for shedding
[18]	10.1186/1471-2334-10-3	Children < 6 y/o, all genders	Diarrhea	RT-PCR (nasopharyngeal and stool)	A and B	Concurrent diarrhea with influenza-like symptoms. 40 patients diagnosed with IBV and 60 with IAV	Recovered
[20]	10.1016/j.jmii.2011.04.003	21 y/o male	Bloody diarrhea, hemorrhagic colitis	Rapid diagnosis kit Capilia Flu A+B, immunochromatography	A	Initially presented with fever and pharyngeal pain later in admission complaining of lower abdominal pain, diarrhea, and hematochezia. Emergency colonoscopy and pathology confirmed hemorrhagic colitis following influenza A	Recovered with zanamivir
[22]	10.1186/1752-1947-5-280	42 y/o female	Nausea, vomiting, severe gastroenteritis	RT-PCR (nasopharyngeal)	A (H1N1)	H1N1-induced hemophagocytic lymphohistiocytosis with GI prodrome	Death due to refractory shock and multi-organ failure
[23]	10.1007/s00104-010-1894-6	15 y/o female	Ischemic colitis	RT-PCR	A (H1N1)	Required colon resection due to ischemia associated with influenza	Death post-surgery
[24]	10.1016/j.pedneo.2013.01.005	14 y/o female	Perforated peptic ulcer, appendicitis	Rapid influenza test	A	Initially presented with abdominal pain, diarrhea, and loss of appetite. Positive influenza test on third day of admission. Oseltamivir started and, 3 days later, had postinfectious peptic ulcer leading to perforation and appendicitis leading to appendectomy	Recovered post-surgery
[25]	10.1136/bcr-2014-208219	9.5 y/o male	Acute appendicitis	RT-PCR	A (H1N1)	Concurrent H1N1 infection and appendicitis	Recovered post-appendectomy
[26]	10.1016/j.hrtlng.2010.04.004	15 y/o female	Acute abdominal pain; suspected appendicitis	RT-PCR	A (H1N1)	Initially presented with appendicitis-like symptoms and influenza-like illness. Appendicitis confirmed on CT ultimately linked to influenza	Recovered

Patient presentations and characteristics derived from case reports regarding gastrointestinal (GI) symptoms/complications with evidence of influenza infection. y/o: years old, RT-PCR: Reverse transcription polymerase chain reaction, GI: gastrointestinal, CT: computed tomography, PCR: polymerase chain reaction.

### 2.3. Routes of Viral Dissemination to GI Tract

The route by which the influenza virus spreads to the GI tract remains debated. One hypothesis suggests that swallowed secretions from a primarily respiratory influenza infection can lead to the infection of the GI lining [11]. Although HA undergoes irreversible conformational Change at acidic pH (<6), rendering the virus inactive, research suggests intact infectivity even after exposure to gastric acid (low pH) [27]. The intact infectivity could be partly attributed to the protection of viral particles by food or viscous mucus secretions, medications like proton-pump inhibitors, or diseases like gastritis and Helicobacter pylori infection that can increase gastric pH. Alternatively, viral mutations can render the viral HA less susceptible to conformational changes in a low pH environment [11,17,28,29,30]. Hematogenous spread of influenza has also been reported in the case of severe avian influenza A infection with high viral RNA loads in blood [31]. However, viral shedding in stool samples without evidence of viraemia suggests that other routes of infection may play a more primary role [11,17,32]. In addition, several reports demonstrate viral entry and replication in myeloid dendritic cells and macrophages, and those infected immune cells may contribute to viral dissemination to various tissues and organs, including the GI tract [33,34].

### 2.4. Pathogenesis of Influenza-Associated GI Symptoms

**Figure 1:**
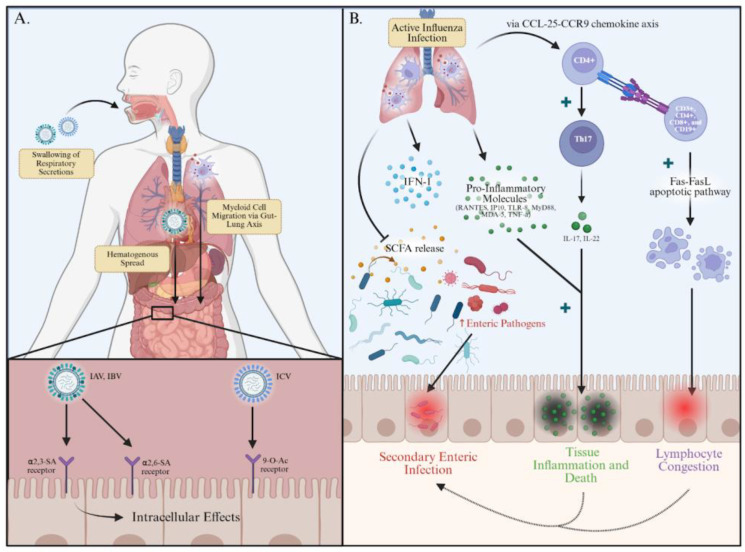
Illustration of the possible mechanisms of gastrointestinal. (GI) symptomatology caused by the influenza virus, through either direct or indirect means. (**A**) Direct Mechanisms of Inflammation: Influenza can disseminate to the GI tract through swallowing of respiratory secretions, hematogenous spread, or infected myeloid cell migration via the gut–lung axis. Once in the GI lumen, HA markers on the surface of IAV/IBV can bind to either α2,3- or α2,6-SA receptors on the surface of intestinal epithelial cells, while HEF markers on the surface of ICV can bind to 9-O-Ac receptors. This leads to viral replication and propagation of disease within the GI tract, causing inflammation and GI symptoms. (**B**) Indirect Mechanisms of Inflammation: During active influenza infection, a variety of effects take place involving the immune system. Influenza virus inhibits the release of SCFAs by gut microbes, encouraging enteric pathogen overgrowth leading to secondary enteric invasion with subsequent infection. The release of IFN-1s, pro-inflammatory markers such as RANTES, IP10, TLR-8, MyD88, MDA-5, and TNF-α, along with increased Th17 replication and subsequent interleukin production, promotes enteric tissue inflammation and apoptosis. Additionally, migratory CD4+ lymphocytes trigger increased apoptosis in CD3+, CD4+, CD8+, and CD19+ cells via the Fas-FasL pathway, which may lead to lymphocyte congestion within enteral tissue. The influx of immunomodulatory molecules, enterocyte inflammation and death, and lymphocyte congestion have the potential to dampen immune system functioning and lead to gut dysbiosis, causing enteral pathogenic invasion. Figures were created in https://www.biorender.com (accessed on 20 June 2025). IAV: Influenza A virus, IBV: Influenza B Virus, ICV: Influenza C Virus, SA: sialic acid, IFN-1: interferon-1, IL: interleukin, TLR: toll-like receptor, TNF: tissue necrosis factor.

The exact pathogenesis of influenza-associated gastroenteritis-like symptoms remain elusive. Here, we categorize the research evidence into either direct or indirect mechanisms of GI inflammation (Figure 1). Direct mechanisms describe viral entry into enteric cells, thereby triggering GI symptoms, while indirect mechanisms outline immunomodulatory alterations and/or gut microbiome dysbiosis as a precipitating factor, which may lead to secondary pathogenic infection.

#### 2.4.1. Direct Mechanisms of Inflammation

For influenza A and B, direct mechanisms involve HA binding to SA receptors, thereby allowing viral entry into host cells. SA receptors can contain α2,3 and α2,6 glycosidic linkages. Avian IAVs exhibit higher specificity for receptors containing α-2,3- glycosidic linkages to SA, while human IAVs preferentially bind to α2,6-linked SA [35]. Similarly, IBV preferentially binds to α2,6 linkages, but certain lineages like the Victoria lineage can bind both α2,3 and α2,6 linkages [36]. The distribution of SA α2,3 linkage and α2,6 linkage varies throughout the body. In humans, the α2,6 linked SA receptors were expressed throughout the GI tract on epithelial cells with increased expression for the α2,3 linked SA receptors from the ileum to the rectum [37]. Similarly, studies in animal models (Pekin ducks and pigs) demonstrated distinct receptor distribution with preferential detection of α2,6 linkages in the duodenum, colon epithelia, and within goblet cells [38,39]. Interestingly, viral RNA of both influenza A and B persisted in stool samples two weeks after clearance from sputum samples (21–28 days from infection), thereby suggesting prolonged GI tract shedding [40]. Successful replication and the production of infectious viral particles were observed using ex vivo cultures of human gut tissues infected with H5N1 virus [37]. Reports of seasonal IAV showed the presence of the virus in antigen-presenting or immune cells in the gut rather than the gut epithelia [41]. GI binding of IBV has not been as thoroughly investigated as influenza A, but it may likely share similar mechanisms to IAV due to comparable entry and replication processes. These findings indicate the possibility of viral binding to SA receptors via α2,3 linkage and α2,6 linkages in the colon, which can potentially lead to inflammation and GI pathology. However, exact viral binding sites remain unclear. 

In contrast, influenza C utilizes HEF glycoprotein to bind N-acetyl-9-O-acetylneuraminic (9-O-Ac) SA receptors for entry and release [6]. Binding of HEF to modified N-acetyl-9-O-acetylneuraminic (9-O-Ac) SA functions as a receptor to allow viral entry while the esterase functions to cleave 9-O-Ac and allow viral release [42,43]. Those modified receptors are found throughout the human GI tract and are abundant in the colon [44]. In human cell lines, 9-O-Ac modifications could be found on the plasma membrane, but they were mostly located in intracellular sites such as the Golgi [38,45,46]. Though research regarding the presence of ICV in the GI tract is limited, the abundant receptor distribution and GI symptoms associated with infection suggest that ICV can directly bind to those receptors and cause GI complications [6,47]. Future studies are still needed to illustrate the molecular mechanisms underlying ICV and GI symptomology.

#### 2.4.2. Indirect Mechanisms of Inflammation

Indirect mechanisms propose that influenza induces GI inflammation via immune dysregulation of GI immunity rather than direct binding to the intestinal epithelia, though both mechanisms may occur concurrently. This complex immune pathway can lead to inflammatory changes through gut microbiome dysbiosis and the upregulation of apoptotic pathways, leading to symptoms of nausea, vomiting, diarrhea, and abdominal pain.

The gut microbiome is integral to human health and has a major impact on immunomodulation and host defense against pathogens [48,49]. Dysbiosis has been associated with conditions like inflammatory bowel diseases, irritable bowel syndrome, celiac disease, and other intra- and extra-intestinal disorders [50]. In addition, dysbiosis has been observed in gastroenteritis caused by norovirus and rotavirus [51]. In a study of 147 influenza patients presenting with diarrhea, mild or moderate dysbiosis was identified in all cases [52]. This shift in microbiome composition was transient and resolved after infection resolution [53]. It has been hypothesized that the influenza virus exploits weakened immune defenses and enables secondary viral or bacterial infections in the GI tract [54]. 

The lung–gut axis plays a critical role in mediating indirect mechanisms of GI inflammation through facilitating immune cell trafficking and immunomodulatory communication between the two organ systems [55]. This axis proposes a bidirectional interaction involving the circulation of inflammatory factors via the bloodstream and lymphatics, a process termed “microbial crosstalk” [56,57]. Short-chain fatty acids (SFCAs), other microbial metabolites, cytokines, and chemokines (including tumor necrosis factor [TNF]-α, transforming growth factor [TGF]-β, interleukins [ILs], C-C-motif chemokine ligands [CCL], C-X-C motif chemokine ligands [CXCL], and RANTES) have all been shown to utilize these pathways [56]. The CCL-25-CCR9 chemokine axis facilitates the translocation of T lymphocytes between the lung and GI tissue through intracellular recognition and signaling. Additional pathways, like IL-33-CXCL16, IL-25-CCL25, and IL-25-CCR9 were also shown to facilitate lymphocytic trafficking utilizing group 2 innate lymphoid cells (ILC2s). Notably, IL-33-CXCL16 signaling was reported to guide ILC2s to the lung tissue, while IL-25-CCL25 signaling guides ILC2s towards intestinal tissue, implying directional complexity within the lung–gut axis [58]. Further, GI microbiota can influence the production of CD4+ and CD8+ T cells in response to influenza infection [56]. Altogether, this implies that both immunomodulatory molecules and immune cells maintain physical interconnectedness between the two organ systems and suggest the role of immune system and microbiome disruption in the development of GI symptoms from influenza infection.

The influenza virus may cause alteration of various immunomodulators like interferons (IFNs), cytokines (including ILs), and leukotrienes, which in turn change gut microbial diversity. For instance, influenza was found to disrupt the gut microbiota, causing an increase in *Escherichia coli* (*E. coli)* and leading to intestinal immune injury in a mouse model, highlighting a role for IFN-γ. Lymphocytes derived from influenza infection in the respiratory mucosa migrated to the intestinal mucosa via a CCL-25-CCR9 chemokine axis. Increased IFN-γ, IL-17A, and IL-15 levels disrupted gut microbiota and promoted Th17-dependent inflammation in the GI tract [59].

Influenza infection prompts the release of type I interferon (IFN-α, -β, and -ω), which has a critical biological role in viral defense mechanisms. Induced IFN-1s in the lungs during acute influenza infection caused the dysregulation of gut microbiota, reduced host immunity, and increased susceptibility to *Salmonella typhimurium* (*S. typhimurium)* [60]. Yildiz et. al., also demonstrated that IAV induced disruption of mucus layer integrity and increased the levels of antimicrobial peptides in Paneth cells, which further facilitated *S. typhimurium* superinfection [61]. Typhoidal salmonella can resist host defense causing fever, fatigue, and GI symptoms [62]. Short-chain fatty acids (SCFAs), produced by symbiotic bacteria in the gut as an end product of fermentation, provide an energy source to enterocytes and control the local pH [63,64]. Influenza virus reduces the production of SCFAs by the microbiota, leading to secondary enteric invasion [65]. For instance, pneumococcal superinfection after influenza virus attenuation has been linked to disruption of gut microbiota and reduced production of SCFAs [66]. Intestinal SCFAs can potentially serve as a biomarker for predicting influenza infection, as the time-course of influenza and SFCA content in GI tissue are linked [67]. Supplemental SCFAs during infection may also be a potential therapeutic option for patients with extensive GI involvement [68]. Branched-chain amino acids (BCAAs), known to contribute to immune imbalance, serve as another possible biomarker as high levels of BCAAs induced by microbiome dysregulation may correlate with a more severe degree of influenza infection [68,69]. Altogether, disruption in the intestinal microbiota and immune damage, mediated via different mechanisms, following influenza could contribute to the enteritis-like symptomatology.

Infection with IAV and IBV results in a dysregulation of cytokines, causing an increase in lymphocyte apoptosis in lymphoid organs, leading to reduced host immunity and exacerbated tissue damage [70,71,72]. IAV has been shown to induce apoptosis, necroptosis, and pyroptosis in host cells, which is altogether termed panoptosis [73,74,75]. In inflammatory bowel diseases, upregulation of proapoptotic features parallels the increasing severity of systemic inflammation, with subsequent bacterial dysbiosis and immune system dysregulation [57,76]. Influenza-induced proapoptotic pathways likely reflect an increase in systemic inflammation.

Interestingly, influenza strains like H9N2 can trigger intestinal epithelium apoptosis through upregulating FAS-L, TNF-α(hundred-fold increase), and other pro-inflammatory molecules (RANTES, IP10, TLR-8, MyD88, and MDA-5), leading to INF-β release [74]. Therefore, similar to GI bacterial pathogens (*Salmonella,* entero-invasive *E. coli, Shigella, Clostridium difficile,* and *Helicobactor pylori*), influenza virus strains can cause intestinal epithelial apoptosis and destabilize the epithelial lining, which consequently contributes to GI symptoms [77].

### 2.5. Other Single-Stranded RNA Viruses

It is worth mentioning that respiratory syncytial virus (RSV) and SARS-CoV-2 virus, both single-stranded RNA viruses, have been associated with GI symptoms.

RSV can disrupt the gut microbiota composition, leading to increased disease severity and increased incidence of asthma, allergies, and lung infections later in life [78,79,80]. A review by Yagi et. al, demonstrated differences in respiratory and gut microbiome between RSV-infected patients and healthy controls [81]. Detection of RSV RNA in stool samples of patients with respiratory illness indicates that GI infiltration is a possible cause of symptomatology [82]. RSV attaches to the host cell through interactions between the viral envelope glycoproteins G or F and cell surface receptors. To facilitate attachment and entry, glycoprotein G binds to cell surface heparan sulfate proteoglycans (HSPGs) and CX3C chemokine receptor 1 (CX3CR1) while glycoprotein F can bind to nucleolin, insulin-like growth factor 1 receptor (IGFR-1), epidermal growth factor (EGF), and intercellular adhesion molecule 1 (ICAM-1) [83]. CX3CR1 has been detected on the apical surface of cultured ciliated human airway epithelia, and the presence of cell surface proteins is limited in human GI tissue. In the GI, ICAM-1 was present on the surface of colon endothelial cells in rat models, and CX3CR1 was identified in intestinal intraluminal dendritic cells [84,85,86].

Accumulating evidence suggests GI infiltration by SARS-CoV-2. A meta-analysis performed in 2020 demonstrated the presence of viral RNA in the stool of 43.7% of SARS-CoV-2-infected patients [87]. Additional studies confirmed the same finding and indicated the prolonged persistence of viral genome in stool for months after respiratory clearance [88,89,90]. SARS-CoV-2 utilizes SA-rich glycoproteins and angiotensin-converting enzyme 2 (ACE2) to facilitate viral entry, indicating the importance of SA in both SARS-CoV-2 and influenza viral infiltration [91]. Transmembrane serine protease 2 (TMPRSS2) facilitates viral entry by cleaving ACE2, thereby allowing transcytosis of the virus [92]. ACE2 and TMPRSS2 are highly expressed in esophageal upper epithelial cells, gland cells, and absorptive enterocytes in the ileum and colon, further implying that SARS-CoV2 has the ability to invade GI tissue [93]. This mechanism is crucial for SARS-CoV-2 entry through the SA-rich mucus layer covering enterocytes in the small intestine [94]. Further, 9-O-Ac has been identified as an attachment factor for coronaviruses to facilitate binding and infection [95].

SARS–CoV-2 infection leads to marked disruption of the gut microbiome, causing further systemic inflammation and immune dysregulation [96]. SARS-CoV-2 was reported to reduce the population of anti-inflammatory bacteria such as *Bifidobacterium* and *Faecalibacterium*, with growth of pathogenic bacteria *Streptococcus, Rothia,* and *Actinomyces* [56]. The degree of gut dysbiosis reflects SARS–CoV-2 severity [97]. This dysbiosis may promote secondary infections through translocation of enteric bacteria into the bloodstream, increasing the mortality of critically ill patients [98,99]. The gut microbiota may also alter ACE2 receptor expression in GI tissue, altering viral binding and facilitating viral entry during active infection. Interestingly, SARS-CoV-2 induced apoptosis in multiple cells within the GI tract, including epithelial, goblet, and lymphoid cells [100,101,102]. However, research elucidating the specific mechanisms by which SARS-CoV-2 mediates the lung–gut axis remains limited, owing in part to the novelty of the gut–lung axis itself as well as its internal complexity [103]. Taken together, pathophysiologic similarities are shared among influenza, RSV, and SARS-CoV-2, indicating possible shared mechanisms of GI infiltration and inflammation among at least these respiratory viruses. 

In all, various RNA viruses can have the capacity to cause GI distress via multiple mechanisms summarized in Table 2.

## 3. Conclusions

Influenza, a highly prevalent respiratory illness, significantly impacts the GI system, causing symptoms ranging from mild illness to more serious complications like colitis. In a population-based study conducted in pediatric IAV-infected patients, 3% among 70 deaths were attributed to GI-related symptoms [104]. In addition, GI-bleeding is an independent predictor for mortality of critically ill influenza patients [105]. Recognizing GI symptoms in influenza infection can improve patient outcome, especially when GI symptoms precede the classic respiratory symptoms. Early diagnosis of these cases can ensure timely administration of proper treatment and better patient prognosis. In addition, early diagnosis and treatment can prevent disease progression to severe morbidity—especially in children, the elderly, and the immunocompromised. Additionally, GI manifestations in influenza infection highlight the potential utility of stool sampling in detection and monitoring of infection. Identification of viral RNA in stool samples can facilitate monitoring the long-term infections in immunocompromised or in patients who are unable to provide sputum samples.

Additionally, high viral shedding in stool samples may have epidemiological concerns in terms of wastewater management. Feces and sputum were shown to be major shedding sources that contributed to SARS-CoV-2 in wastewater demonstrating these sources to be potentially viable in spread through wastewater [106]. In Germany, IAV was detected in 5.2% and 41.6% of samples in wastewater treatment plant 1 and plant 2, respectively, while IBV was found in 36.0% and 57.7% of samples, respectively [107]. This underscores concerns for potential spread of influenza infection and the need for wastewater surveillance. In Ottowa, wastewater sampling enabled the detection of IAV 17 days prior to its clinical detection by conventional methods in hospitals or clinics during outbreak in 2022 [108]. This demonstrates that GI manifestations and viral shedding via the GI tract can support earlier clinical detection and public health improvement, thus allowing more proper and timely allocation of resources during outbreaks.

Future research should evaluate the effectiveness of clinical markers, like calprotectin or SCFAs of microbiota, for assessing the disease severity or risk of secondary infections. This will be particularly important for pediatric and immunocompromised populations, which often have prolonged viral shedding, higher rates of intestinal cell death, and weaker mucosal immunity. In addition, another area of research is to determine whether probiotics or postbiotics can possibly aid in restoring the microbiome and lowering GI inflammation during or after influenza or other viral infection. Further, controlled studies are required in the future to investigate the value of early antiviral treatment in patients with severe GI manifestations. Future studies should evaluate the association between GI severity profile with the various subtypes of influenza infection. This will enable proper clinical management which may include GI protection to decrease the likelihood of severe GI complications such as colitis. Understanding the exact pathogenesis of GI involvement by specific viral subtypes can enable the development of targeted pharmacological intervention to decrease GI severity. Given that most studies screened were observational with a higher predominance of IAV, there is still a need to determine causality of influenza infection and GI manifestation as well as investigating the impact of IBV and ICV on the severity of GI manifestations. Discerning direct or indirect inflammatory pathways may equip physicians to understand GI dominant presentations and better manage possible complications. Understanding the connection between the GI system and respiratory viral infection can lead to new clinical approaches, better patient outcomes, and updated public healthcare policies designed for at-risk populations.

## Figures and Tables

**Table 2:** Comparative summary of virological and GI clinical features of influenza and other common respiratory viruses.

Virus	Receptor	GI Entry Evidence	Stool Shedding	Major Clinical GI Symptoms	Potential Direct Mechanisms	Potential Indirect Mechanisms
IAV	α2,6-SA	Strong	Yes	Diarrhea, nausea, emesis, acute abdomen, hemorrhagic colitis, appendicitis	Binding of HA α2,6- and α2,3 glycosidic linkages to α2,6- and α2,3-SA receptors, respectively, allowing for viral entry and inflammation. Preference for binding to α2,6-SA receptors.	Gut microbiome dysbiosis Upregulation of proinflammatory molecules and apoptosis causing tissue inflammation and death Lymphocytic congestion Secondary enteric invasion by pathogenic gut bacteria
IBV	α2,6-SA	Moderate	Yes	Nausea, diarrhea	Binding of HA α2,6- and α2,3 glycosidic linkages to α2,6- and α2,3-SA receptors, respectively, allowing for viral entry and inflammation. Mostly prefer binding to α2,6-SA receptors. Victoria-lineage preference for α2,3-SA.	Gut microbiome dysbiosis Upregulation of proinflammatory molecules Lymphocytic congestion
ICV	9-O-Ac-SA	Limited	Not well studied	Nausea, diarrhea	Binding of HEF glycoprotein to 9-O-Ac SA viral entry. Esterase of HEF cleaves 9-O-Ac to allow for viral release.	Not well studied
RSV	Multiple: HSPGs, CX3SR1, nucleolin, IGFR-1, EGF, ICAM-1	Moderate	Yes	Diarrhea, altered gut microbiome	Binding of glycoprotein G to HSPGs and CX3CR1. Binding of glycoprotein F to nucleolin, IGFR-1, EGF, and ICAM-1. Limited application of direct binding. Distribution of cell surface markers and RSV binding in animal models.	Gut microbiome dysbiosis
SARS-CoV-2	ACE2 and SA-rich glycans	Strong	Yes	Diarrhea, nausea, GI bleeding	TMPRSS2 cleaving of ACE2 9-O-Ac potential attachment factor for binding	Epithelial, goblet, and lymphoid cell apoptosis. Marked gut microbiome disruption with ACE2 receptor expression dysregulation. Specific mechanisms of inflammation via the lung–gut axis are unknown.

Summary of key findings on GI symptoms and possible pathogenesis based on current research for influenza, RSV, and SARS-CoV-2. IAV: Influenza A virus, IBV: Influenza B Virus, ICV: Influenza C Virus, SA: sialic acid, HSPG: heparan sulfate proteoglycans, CX3CR1: CX3C chemokine receptor, IGFR-1: insulin-like growth factor 1 receptor, EGF: epidermal growth factor, ICAM-1: intercellular adhesion molecule 1, ACE2: angiotensin converting enzyme 2, GI: gastrointestinal, HA: hemagglutinin, 9-O-Ac: N-acetyl-9-O-acetylneuraminic, TMPRSS2: transmembrane serine protease 2.

## Abbreviations

The following abbreviations are used in this manuscript:

GI	Gastrointestinal
CDC	Centers of Disease Control and Prevention
WHO	World Health Organization
IAV	Influenza A
IBV	Influenza B
ICV	Influenza C
IDV	Influenza D
SA	Sialic acid
HA	Hemagglutinin
NA	Neuraminidase
RNA	Ribonucleic acid
HEF	Hemagglutinin-esterase-fusion
9-O-Ac	N-acetyl-9-O-acetylneuraminic
Y/o	Years old
RT-PCR	Reverse transcription polymerase chain reaction
CT	Computed tomography
PCR	Polymerase chain reaction
IFN	Interferon
IL	Interleukins
TLR	Toll-like receptor
TGF	Transforming growth factor
TNF	Tissue necrosis factor
CCL	C-C-motif chemokine ligands
CXCL	C-X-C motif chemokine ligands
ILC2	Group 2 innate lymphoid cells
SCFA	Short-chain fatty acids
BCAA	Branched-chain amino acids
RSV	Respiratory syncytial virus
HSPG	Heparan sulfate proteoglycans
CX3CR1	CX3C chemokine receptor 1
IGFR-1	Insulin-like growth factor 1 receptor
EGF	Epidermal growth factor
ICAM-1	Intercellular adhesion molecule 1
SARS-CoV-2	Coronavirus
ACE2	Angiotensin converting enzyme 2
TMPRSS2	Transmembrane serine protease 2
